# Useful Medication in Typhoid Fever

**Published:** 1896-11

**Authors:** Henry R. Slack

**Affiliations:** LaGrange, Ga.; Professor of Chemistry in Southern Female College, Member of the American Medical Association, Secretary Georgia State Board of Pharmacy, Etc.


### USEFUL MEDICATION IN TYPHOID FEVER.

By HENRY R. SLACK, Ph.M., M.D., LaGrange, Ga.

Professor of Chemistry in Southern Female College, Member of the American Medical Association, Secretary Georgia State Board of Pharmacy, Etc.

Years ago I remember hearing the late learned and beloved Dr. H. V. M. Miller say in a lecture on typhoid fever:

"Young gentlemen, when I practiced medicine up in the mountains, I had much typhoid fever to treat. My territory was very large, and many of my patients I did not see but three or four times during the course of the disease. On being called for, the first time I usually found my patient with pains in the back and limbs, headache, loss of appetite, coated tongue, flushed face and fever. I can't give you the temperature, for in those days the clinical thermometer was not used. I gave a good dose of calomel, saw that they had a bottle of turpentine, inquired about the supply of milk, but seldom asked about whisky, for of that there was always an abundance. I gave directions about diet, the use of cold water when temperature rose (the coal-tar antipyretics were then unknown), and turpentine if the bowels became tender or tympanitic, and whisky if the pulse became feeble or collapse was threatened. I then left, with instructions to send for me if any complications came up. Looking over my cases, I find that I lost only a little over eight per cent. of my typhoid fever patients.

"When I moved from the mountains to the more thickly settled country, where the doctors saw their patients every day, I noticed that the mortality increased to twelve or fifteen per cent., and some of the very active doctors *saved* eighty per cent. of their typhoid fever patients by active medication.

Last winter, while at the Johns Hopkins Hospital, I heard that prince of clinicians, Dr. Osler, say to his ward class while standing beside a typical case of typhoid fever: "Gentlemen, about eight per cent. of all such cases are doomed—they have been called. About eight more depend upon the doctor and nurse for life, principally the nurse; four more will stand a good deal of doctoring, and eighty per cent. will recover in spite of the doctor, unless he uses the coal-tar antipyretics too freely."

Now, with such authorities and many others against medication, it may seem almost presumptuous to start upon a regular system of medication, but Dr. John E. Woodbridge has had the temerity to work persistently on this line, and, judging from his frequent reports to the various medical associations and articles in the *Journal of the American Medical Association,* his efforts have been crowned with success.

We are told in the Scriptures to "Prove all things, hold fast to that which is good," and this is one precept of the Bible, I believe we all try to follow, though what seems "strong as proof of Holy Writ" to some minds, appears to others "trifles light as air."

It was my good fortune to have served ten years as pharmacist before studying medicine, and during that time, and since, to be intimately associated with Dr. Enoch Callaway, one of the most accomplished therapists and successful general practitioners I have ever seen. Together we have worked out many new combinations of drugs, he furnishing the therapeutics and I the chemistry necessary for their proper preparation. He made the following prescription to use in a case of typhoid fever, where the temperature was very high, but the heart complications rendered the use of the usual antipyretics dangerous :

℞ Ammonii carbonatis........................................ 8 gm. (ʒ ii.)
Acidi salicylici.............................................10 gm. (ʒ iiss.)
Elix. pepsini lactat.,
Aquæ cinnamomi, āā .......................................60 cc. (℥ ii.)
M. Sig.--Teaspoonful in a little water every three hours.

Here we get the antipyretic and antiseptic effects of the salicylate and at the same time the stimulation of the ammonium carbonate. The lactated pepsin and cinnamon water enable the stomach better to tolerate the salicylate and prevent it interfering with the digestion. Experience now with 100 cases* shows a marked diminution in complications and unpleasant sequelæ. Tympanites, hyperpyrexia, diarrhea, and nervous symptoms are much

* The majority of these cases occurred in the practice of my colleagues, Drs. E. Callaway and E. A. Cason.

less frequent than before the use of this prescription, and only three deaths in 100 cases covering five years; so that it cannot all be attributed simply to a mild epidemic.

My experience with the Woodbridge treatment, while fairly satisfactory, has been that the temperature does not fall after the bowels are moved, as claimed by its originator, but by supplementing it with the elixir salicylate ammonia, I have little trouble with hyperpyrexia.

Now, I don't claim to avert typhoid fever by the use of this prescription, but I know, from clinical experience, it does relieve the patient of many distressing symptoms from which they formerly suffered; and, in combination with Woodbridge treatment Nos. 1 and 2, the fever assumes the mild or abortive type. In 66 per cent. of the cases where used, the temperature has reached normal by the fifteenth day.

A word about having this prescription filled. Be sure to caution your pharmacist to use only the translucent lumps of ammonium carbonate. Dissolve them in the cinnamon water, then add the salicylic acid, and lastly the elixir of lactated pepsin.

